# Knowledge, awareness, and perception of digital dentistry among Egyptian dentists: a cross-sectional study

**DOI:** 10.1186/s12903-023-03698-1

**Published:** 2023-12-04

**Authors:** Mohamed Ashraf Hall, Inas Karawia, Ahmed Zakaria Mahmoud, Osama Safwat Mohamed

**Affiliations:** 1 Alexandria Dental Research Center, Ministry of Health and Population, Alexandria, Egypt; 2 Pediatric and Community Dentistry Department, Faculty of Dentistry, Pharos University, Alexandria, Egypt; 3 Ministry of Health and Population, Alexandria, Egypt; 4 Department of Prosthodontics, College of Dentistry, Arab Academy for Science, Technology, and Maritime Transport, El Alamein, Egypt; 5 Dental Prosthesis Manufacture Technology Department, Faculty of Applied Health Sciences Technology, Pharos University, Alexandria, Egypt

**Keywords:** Knowledge, Awareness, Perception, Digital dentistry, Dentist, Egypt

## Abstract

**Background:**

Digital dentistry has revolutionized the way dental treatment is offered to patients. It became essential for dental practitioners to be well-informed about this technology to improve the quality of care offered and increase patient satisfaction. This study aimed to assess the level of knowledge, awareness, and perception of Egyptian dentists toward digital dentistry.

**Methods:**

An online-based cross-sectional study was conducted using social media platforms from November 2022 to March 2023. Our study sample included dentists with different levels of experience, specialties, and working in different health sectors in Egypt. A questionnaire arranged in 4 sections and 23 questions was used as the study data collection tool. The data were tabulated and analyzed using SPSS software.

**Results:**

A total of 402 participants filled out this questionnaire. 50.7% of which were females, 42.8% were between 20–29 years old and 42.3% were general practitioners. Furthermore, the main practice of 27.6% was in governmental dental clinics. Moreover, 47.3% and 64.2% of participants had Moderate knowledge and awareness respectively. While 75.9% of them had a high perception of practicing digital dentistry. Females and practitioners in governmental clinics had significantly lower awareness scores, while faculty teaching staff had significantly higher scores (*P* ≤ 0.05). On the other hand, practicing in the Great Cairo region and urban areas was associated with significantly higher knowledge scores (*P* ≤ 0.05). Similarly, Prosthodontists, periodontists, and restorative dentists had significantly higher scores when compared with general dentists (*P* ≤ 0.05).

**Conclusions:**

About half of the study participants had Moderate knowledge and awareness levels, while about three-quarters of them had a high level of perception toward practicing digital dentistry. Therefore, more attention should be given to providing dental education programs in this important field at both the undergraduate and postgraduate levels by policymakers.

**Supplementary Information:**

The online version contains supplementary material available at 10.1186/s12903-023-03698-1.

## Introduction

Modern dentistry has changed a lot from what was previously taught in dental schools. The recent development of digital dental tools signifies not just a technological innovation but also a fundamental reorganization of healthcare systems, encompassing everything from patient-doctor communication to treatment procedures. Since the introduction of digital dentistry in our practices, there has been a paradigm shift in the way dental treatments are offered to patients [1]. With current equipment, x-ray shifted from 2D to 3D, intra-oral scanning (IOS) is used instead of conventional impression materials, prosthetic and implant software, along with milling machines and 3D printers, are used to create esthetic and precise restorations in a relatively short time [2], which in turn increased the efficacy and the patient’s satisfaction [3]. The main basic phases of the digital workflow are data acquisition, data processing, and the manufacturing phase. Digital photography, cone beam computed tomography (CBCT), and optical scanning are used in the first phase to capture the patient data. While data is processed using different computer-aided design (CAD) softwares, which have different modules for different specialties. The manufacturing phase is divided into subtractive or additive techniques with milling machines or 3D printers, respectively [2].

Almost all dental specialties adopted the digital workflow from the point of diagnosis till the provision of the final treatment [2]. Inlays, onlays, veneers, and crowns can be delivered to the patients on the same day, which increases patient satisfaction [4]. Similarly, in removable prosthodontics, the complete denture workflow is completed with comparable success to conventional approaches [5]. The use of CBCT and 3D printing technology [6], has resulted in a significant improvement in the precision and total control over the quality of treatment in various dental operations through the use of surgical guides. Prior to dental implant surgery, CBCT is used in the initial step of diagnosis to give an accurate estimation of the bone volume and position of the anatomical landmarks [7], while the surgical guides are fabricated to ensure an accurate position of the dental implants in the arch for optimal performance of the prosthetic part [8]. Moreover, surgical guides can be used in endodontics to aid in obliterated canal detection, endodontic microsurgery, and guided auto transplantation which constitutes a major advance in this field [9]. Furthermore, digital technology allows the fabrication of clear aligners, customized appliances, and retainers in orthodontics, which in turn enhances the appliance’s precision, overall treatment time, and predictability [10]. While in maxillofacial surgeries, patient-specific implants, surgical guides, and skeletal reconstruction are among the most important uses of this technology [11]. This increase in precision and patient satisfaction that comes with digital dentistry technology’s infiltration into several dental disciplines requires greater knowledge and awareness of this technology among practicing dentists.

In addition to its clinical advantages in several dental disciplines, a review in 2021 concluded that the digital dental workflow contributed to increased safety and reduced transmission of the COVID-19 virus. This is achieved by reducing the length and the number of dental appointments, less invasive surgical procedures, and reduced contact between the lab and the clinic [12]. However, with all the advantages that come with modern dental technology, the high cost of the equipment, lack of standardized workflow, the size of some intra-oral scanners, and the learning curve are some of the shortcomings of digital technology [13]. Considering the vast advantages and development of digital dental equipment, with relatively few drawbacks. It became necessary to increase awareness and knowledge about its application in our field of practice.

To assess the knowledge and practice of digital dentistry across the globe, surveys were made in some countries. In 2016, a survey was done in the UK, and it concluded that 55.6% of the respondents didn’t use digital dentistry due to the high cost [14], while in the Netherlands the use was much higher, especially among practice owners [15]. In India, a study among dental practitioners found that 96.7% were aware of CAD/CAM technology in dentistry, and 87% believed that lack of knowledge was one of the shortcomings of CAD-CAM rather than its high cost [16], while another study concluded that 74% of undergraduate students were unaware of the materials used to fabricate the CAD-CAM prosthesis [17]. Similarly, studies in Saudi Arabia showed that the majority of the individuals (98.5%) believed that digital dentistry improved the quality of dental procedures and would eventually replace traditional dental services [18, 19].

In Egypt, the market for digital dental technology has grown noticeably, especially in private dental practices, even though the majority of dentists in Egypt work in the governmental sector [20]. This shows that practitioners in various institutions may differ in their utilization patterns and, consequently, in their level of knowledge. When compared to other developed countries, the primary barrier to market expansion in Egypt continues to be the high cost of digital technology [21]. Up to the author’s knowledge, no study was found to assess dental digital technology knowledge among Egyptian dentists. The null hypothesis of the underlying study is that there is no significant difference in Egyptian dentists’ knowledge, awareness, and perception (KAP) of digital dentistry across specializations and institutions. This study aimed to assess (KAP) of digital dentistry among dental practitioners with different specialties and in different institutions (private, governmental, and academic sectors) in Egypt.

## Methods

### Study design and ethical considerations

A cross-sectional study which is based on an online questionnaire using Google form was conducted between November 2022 and March 2023. The study was conducted after approval of the research ethics committee at Pharos University registration no (04–2022-11–27-3–047). The objective of the study was explained to the participants through a cover letter at the start of the Google form, stating that participation was voluntary and anonymous, and the estimated time required for filling the form. Written informed consent was obtained prior to the completion of the questionnaire. The study followed the (STROBE) guidelines for reporting observational studies.

### Sample size and sampling method

The link to the Google form was sent to the participants using social media networks (WhatsApp, Telegram, and Facebook groups). The snowball sampling technique was used, where the participant was asked to send the form to their friends and colleagues to make sure our sample was as representative as possible. General and specialist dentists working in the governmental, private, and academic sectors were included in the study. While interns and undergraduate students were excluded. Based on an estimated percentage of dentists’ knowledge about digital dentistry of 50% and precision of 5%. The minimum required sample was calculated to be 385 participants using the Epitools at a 95% confidence level.

### Data collection tool

A self-administered, questionnaire with 23 questions (Appendix 1) in the English language based on relevant literature [16] was adopted with some changes in the demographic characteristics and the addition of the “I don’t know” answers to the knowledge and awareness questions. The first sections included demographic characteristics (age group, years of experience, type of practice, governorate of practice, and specialty). The second section consisted of five questions that assessed the participants’ knowledge of the uses, advantages, and shortcomings of CAD-CAM technology. The third section consisted of four questions that assessed the level of awareness of CAD-CAM technology in clinical applications, uses, common CAD-CAM systems, and materials used with it. For the knowledge and awareness section, each correct answer was scored with 1 point while incorrect answers including “I don’t know”, “None” and “No” received 0 points. The fourth section examined the perception and practices of the study participants, and it consisted of seven questions, with a score of 2 for “yes”, 1 for “No” and 0 for “Not sure”. The responses were set to one response to prevent multiple entries. The participants were evaluated according to the following scores:

- ***Knowledge Scores***: Low: 0–8, Moderate: 9–17, and High: 18–26
- ***Awareness Scores***: Low: 0–3, Moderate: 4–7, and High: 8–10
- ***Perception Scores***: Low: 0–4, Moderate: 5–9, and High: 10–14

The questionnaire was re-assessed by sending it to a panel of experts to ensure the quality of the data to verify its content validity. The questionnaire was pilot-tested on 45 participants to ensure the clarity of the questions. The reliability was checked and the Cronbach’s alpha was 0.716.

### Study outcome

The primary outcome was knowledge, awareness, and perception which were analyzed depending on the participant’s demographic variables.

### Statistical analysis

The collected data were revised, coded, and analyzed using SPSS version 25 software for tabulation and analysis. The significance of the obtained results was judged at a 5% level. Categorical variables were summarized by frequency and percent. Linear regression was used to examine the association between sociodemographic factors and knowledge, awareness, and perception mean values.

## Results

As shown in (Table 1), 402 participants responded to this questionnaire. 49.3% of the participants were males and 50.7% were females. Regarding the distribution of participants by their age it was found that 42.8%, 40.8%, 12.9%, and 3.5% were between 20–29, 30–39, 40–49, and 50–59 respectively. The main practice of 25.1% of participants was in private clinics, 27.6% were working mainly in governmental dental clinics, 18.7% were faculty teaching and 28.6% were postgraduate students. 36.1% of participants had less than 5 years of experience, while 28.6% had between 5–10 years and 35.3% had more than 10 years. Concerning governorate of practice, 28.6% of participants were practicing in Great Cairo, 51.7% in Alexandria, 13.4% in Delta and Suez Canal, and 6.2% were practicing in Upper Egypt. Although, it was found that 87.1% were practicing in urban areas and 12.9% in the rural area. It was noticed that 42.3%, 6.0%, 19.4%, 1.0%, 11.4%, 3.7%, 8.0%, 6.0%, and 2.2% were general practitioner dentists, family dentists, prosthodontists, orthodontists, oral surgeons, restorative dentists, pedodontists, periodontists, and preventive dentists respectively.

**Table 1:** Distribution of the participant by their demographic criteria

	**No. (*n* = 402)**	**%**
**Gender**		
Male	198	49.3
Female	204	50.7
**Age**		
20–29	172	42.8
30–39	164	40.8
40–49	52	12.9
50–59	14	3.5
**Main practice**		
Private	101	25.1
Governmental	111	27.6
Faculty Teaching	75	18.7
Postgraduate Student	115	28.6
**Years of Experience**		
Less than 5	145	36.1
5–10	115	28.6
More than 10	142	35.3
**Governorate of Practice**		
Great Cairo	115	28.6
Alexandria	208	51.7
Delta/ Suez Canal	54	13.4
Upper Egypt	25	6.2
**Location of Practice**		
Urban	350	87.1
Rural	52	12.9
**Specialty**		
General practitioner	170	42.3
Family Dentist	24	6.0
Prosthodontist	78	19.4
Orthodontist	4	1.0
Oral Surgeon	46	11.4
Restorative	15	3.7
Pedodontist	32	8.0
Periodontist	24	6.0
Preventive dentist	9	2.2

**Fig. 1:**
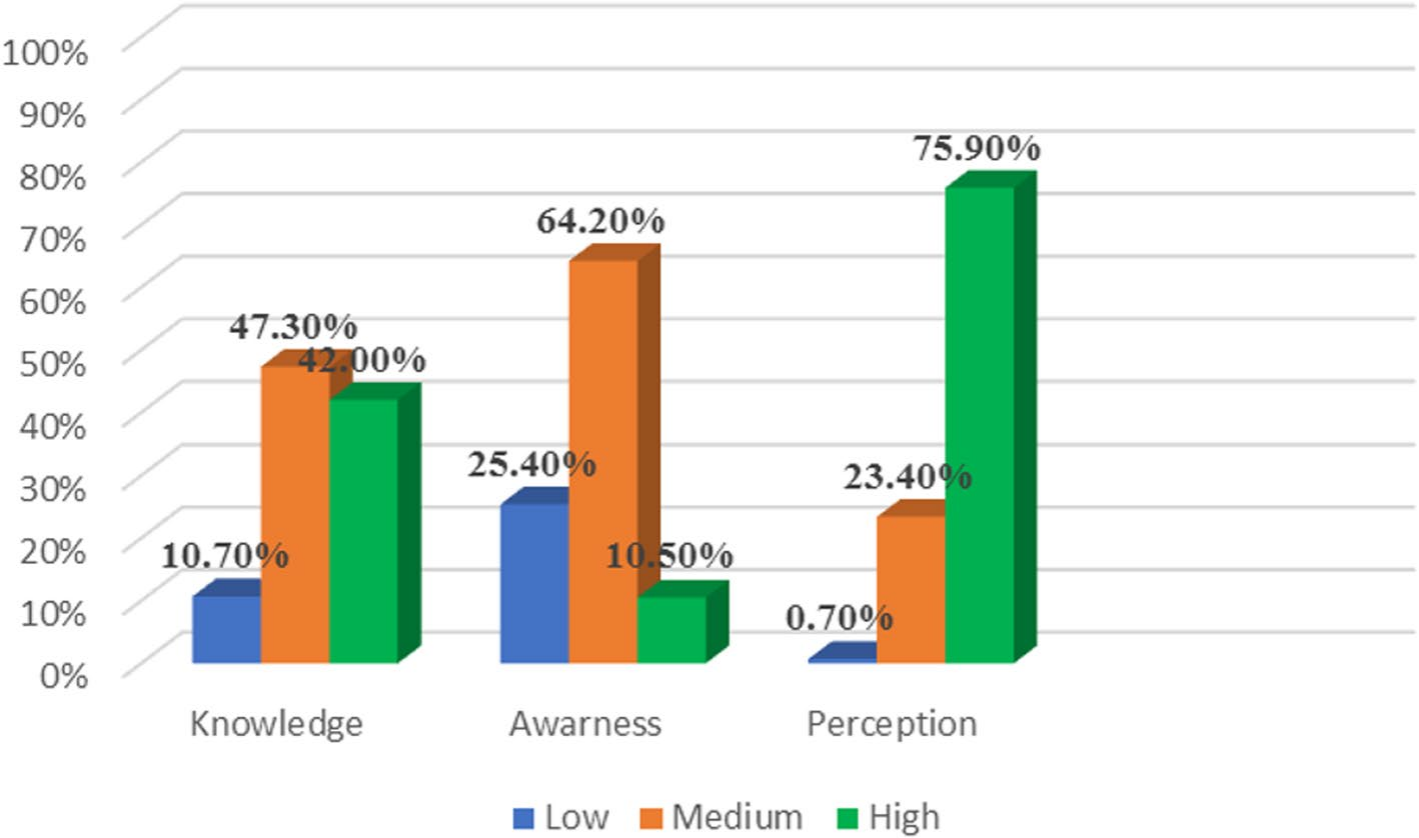
Distribution of the participants regarding their level of knowledge, awareness, and perception

Figure 1 shows that 47.3% and 64.2% of participants had Moderate knowledge and awareness respectively. While 75.9% of them had a high perception toward practicing digital dentistry.

Multivariate linear regression analysis (Table 2) shows that in comparison to the male gender regarding awareness and perception [5.3(2.1) and 11.0(1.9)], females had significantly lower scores [4.4(2.2) and 10.3(2.3)] (*p* = 0.001 and 0.042) respectively. Participants who were practicing dentistry mainly in governmental clinics had significantly lower awareness scores [4.1(2.1)], while faculty teaching staff had significantly higher scores [5.7(2.3)] when compared with those who had their main practice in private clinics [4.7(2.3)], (*p* = 0.003 and 0.029 respectively). In comparison with knowledge score of participants taking Great Cairo as governorate of practice [17.3(4.8)], Alexandria [15.4(5.7)] and Delta/Suez Canal [14.4(4.9)] were significantly lower (*p* = 0.002 and 0.001) respectively, but in comparison with perception score of Great Cairo participants [10.7(2.2)], only Delta/Suez Canal [9.9(2.3)] showed significant difference (*p* = 0.029). Participants who were practicing in rural areas had significantly lower knowledge, awareness, and perception [13.4(4.4), 3.9(1.9), and 9.8(1.9)] when compared with those who were practicing in urban areas [ 16.3(5.4), 5.0(2.2) and 10.7(2.1)] (*p* = 0.004, 0.003 and 0.001) respectively. When comparing knowledge scores with general practitioner dentists [14.9(5.1)], it was found that prosthodontists and periodontists [19.7(4.6) and 17.8(3.5)] were significantly higher (*p* = 0.000 and 0.007) respectively, however, pedodontists [12.8(3.9)] and preventive dentists [9.7(3.8)] were significantly lower (*p* = 0.035 and 0.011) respectively. In comparison with the awareness score of general practitioner dentists [4.4(2.0)], it was found that prosthodontists [6.3(2.1)] and restorative dentists [6.9(3.3)] significantly had higher scores (*p* = 0.000), while preventive dentist showed significantly lower scores [2.1(1.5)] (*p* = 0.004). Pedodontists [8.2(2.7)] and preventive dentists [7.0(2.5)] had significantly lower perception scores when compared with general practitioner dentists [10.7(1.9)] (*p* = 0.000). There were no significant differences regarding age and years of experience factors (*p* > 0.05).

**Table 2:** Multivariate linear regression analysis of knowledge, awareness, and perception regarding digital dentistry

	Total Knowledge			Total Awareness			Total Perception		
	Mean(SD)	ß[95% CI]	*P* value	Mean(SD)	ß[95% CI]	*P* value	Mean(SD)	ß[95% CI]	*P* value
**Gender**									
*Male((Ref)*	16.7(4.9)			5.3(2.1)			11.0(1.9)		
Female	15.4(5.7)	-0.94[-1.9–0.05]	0.062	4.4(2.2)	-0.67[-1.06–0.27]	0.001^*^	10.3(2.3)	-0.416[-0.82–0.02]	0.042^*^
**Age**									
*20–29(Ref)*	16.4(4.7)			4.9(1.9)			10.9(2.0)		
30–39	15.4(5.8)	-0.73[-1.93–0.05]	0.389	4.8(1.9)	0.03[-0.63- -0.69]	0.931	10.5(2.2)	-0.30[-0.96–0.38]	0.395
40–49	17.3(5.2)	0.99[-1.39–3.36]	0.416	4.8(1.8)	-0.18[-1.13–0.77]	0.707	10.5(1.7)	-0.53[-1.49–0.43]	0.281
50–59	13.9(7.0)	-2.13[-5.28- -1.02]	0.185	3.9(2.3)	-1.20[-2.5–0.05]	0.060	9.5(3.1)	-1.041[-2.31–0.23]	0.108
**Main practice**									
*Private (Ref)*	15.5(4.5)			4.7(2.3)			10.7(2.2)		
Governmental	14.9(6.0)	-1.02[-2.34–0.31]	0.131	4.1(2.1)	-0.79[-1.31- -0.26]	0.003^*^	10.7(1.9)	-0.03[-0.57–0.50]	0.907
Faculty Teaching	17.7(5.8)	1.51[-0.03–3.05]	0.055	5.7(2.3)	0.68[0.07–1.30]	0.029^*^	10.2(2.4)	-0.24[-0.86–0.39]	0.457
Postgraduate Student	16.3(4.7)	-0.32[-1.77–1.13]	0.663	5.1(1.9)	-0.014[-0.59-.56]	0.963	10.8(2.2)	0.01[-0.57–0.59]	0.968
**Years of Experience**									
*Less than 5 (Ref)*	16.2(4.7)			4.9(1.9)			10.8(2.1)		
5–10	16.3(5.2)	-0.12[-1.72–1.48]	0.884	5.0(2.4)	-0.07[-0.71–0.57]	0.832	10.8(2.1)	0.24[-0.40–0.89]	0.460
More than 10	15.5(6.1)	-1.46[-3.57–0.65]	0.174	4.6(2.3)	-0.33[-1.17–0.51]	0.440	10.4(2.1)	0.04[-0.81–0.89]	0.930
**Governorate of Practice**									
*Great Cairo* Region *(Ref)*	17.3(4.8)			5.7(2.3)			10.7(2.2)		
Alexandria Region	15.4(5.7)	-1.90[-3.10- -0.70]	0.002^*^	4.4(1.9)	-1.27[-1.75- -0.79]	0.000^*^	10.8(2.1)	0.02[-0.47–0.50]	0.952
Delta/Suez Canal	14.4(4.9)	-2.85[-4.55- -1.15]	0.001^*^	4.3(2.5)	-1.41[-2.09- -0.73]	0.000^*^	9.9(2.3)	-0.77[-1.46- -0.08]	0.029^*^
Upper Egypt	18.4(3.8)	1.10[-1.17–3.38]	0.341	5.5(2.35)	-0.17[-1.08–0.75]	0.720	10.6(1.5)	-0.09[-1.01–0.83]	0.847
**Location of Practice**									
*Urban (Ref)*	16.3(5.4)			5.0(2.2)			10.7(2.1)		
Rural	13.4(4.4)	-2.18[-3.65- -0.71]	0.004^*^	3.9(1.9)	-0.90[-1.49- -0.32]	0.003^*^	9.8(1.9)	-1.02[-1.61- -0.42]	0.001^*^
**Specialty**									
*General (Ref)*	14.9(5.1)			4.4(2.0)			10.7(1.9)		
Family Dentist	15.3(6.1)	1.22[-0.92–3.36]	0.262	4.2(2.0)	0.04[-0.82–0.89]	0.934	10.6(2.1)	-0.01[-0.88–0.85]	0.977
Prosthodontist	19.7(4.6)	4.62[3.16–6.09]	0.000^*^	6.3(2.1)	1.68[1.09–2.26]	0.000^*^	11.4(1.6)	0.59[-0.002–1.18]	0.051
Orthodontist	17.5(7.7)	2.15[-2.75–7.05]	0.388	5.8(2.6)	1.33[-0.62–3.28]	0.180	11.5(1.0)	0.77[-1.20–2.75]	0.442
Oral Surgeon	16.2(4.8)	1.61[-0.29–1.11]	0.072	4.8(1.5)	0.41[-0.29–1.11]	0.252	11.0(1.8)	0.33[-0.38–1.04	0.360
Restorative Dentist	16.9(6.0)	1.70[-1.06–4.45]	0.227	6.9(3.3)	2.38[1.28–3.48]	0.000^*^	11.0(1.5)	0.444[-0.67–1.56]	0.433
Pedodontist	12.8(3.9)	-2.11[-4.07- -.14]	0.035^*^	3.9(1.9)	1.68[-1.47–0.09]	0.083	8.2(2.7)	-2.32[-3.11- -1.53]	.000^*^
Periodontist	17.8(3.5)	3.08[0.84–5.33]	0.007^*^	4.5(2.0)	0.21[-0.68–1.10]	0.646	11.0(1.6)	0.49[-0.42–1.39]	0.290
Preventive dentist	9.7(3.8)	-4.45[-7.87- -1.03]	0.011^*^	2.1(1.5)	-1.98 [-3.34- -0.62]	0.004^*^	7.0(2.5)	-3.29[-0.42–1.39]	0.000^*^

*Ref* The reference

^*^Significance difference ≤ 0.05

## Discussion

The digital revolution is changing the world, without the exception of the dental field. It is important for dentists to have a better understanding and knowledge about the new trend. This study aimed to address the lack of literature regarding dentists’ knowledge, awareness, and perception of digital dentistry in Egypt, this would give us better information to be able to direct continuous dental education in this important field effectively. The findings of our study revealed a substantial difference among participants across specializations, regions of practice, and institutions, so the null hypothesis can be rejected.

In addition to the increased precision and comfort associated with the use of digital dentistry for practitioners, patient satisfaction has been reported in several studies. A survey conducted in Italy on patients’ and parents’ acceptance of clear orthodontic aligner therapy, showed more improvement in the patient’s social and school lives, increased overall satisfaction, and less painful to wear than traditional interceptive orthodontics [22]. While, according to the finding of a clinical trial conducted in Turkey (2014), the digital impression technique was more efficient, with a shortening of the treatment time, and more accepted from the patient’s point of view than the conventional impression technique [3]. Similar findings were also revealed by a survey conducted in the United States by Saponaro PC et al. [23] among a group of experienced complete denture wearers who confirmed a positive satisfaction rating with their digitally fabricated complete dentures in comparison with traditionally fabricated ones. Increased patient satisfaction with digital dentistry results, direct practicing dentists to be more knowledgeable and start adopting this technology.

In a survey conducted by Ramesh Nayakar et al. [16] in India (2022), 96.7% of the study participants were aware of CAD/CAM technology in dentistry. These findings are in disagreement with the results of the current study where 47.3% and 64.2% of participants had Moderate knowledge and awareness respectively, while 75.9% of them had a high perception of practicing digital dentistry. This may be due to the fact that more than half of the study participants in the Indian study were postgraduate students or the difference in the dental curriculum between the two countries. Furthermore, females had considerably lower awareness and perception scores in the current study as compared to males, which may be due to male practitioners’ preference for fixed prosthodontics as a specialization, according to a study conducted among dental students in Egypt [24]. This is inconsistent with a study conducted in Saudi Arabia which found that females showed higher results with most of the knowledge, awareness, and perception questions about digital dentistry [18]. Mostly, the high level of knowledge among Saudi Arabia’s interns and postgraduate students in India may be attributed to the use of digital education in conjunction with accessibility, collaboration, and communication between instructors and students, which support the transfer of theoretical and practical knowledge.

In Egypt, dental care is provided by three main sectors: governmental, private, and academic healthcare facilities, with most of the dentists practicing in the governmental sector [20]. When comparing dental practitioners in these sectors, the findings of the present study concluded that governmental clinics participants had significantly lower awareness scores, while faculty teaching staff had significantly higher awareness when compared with those who had their main practice in private clinics. Which highlights a significant gap in continued professional training among the participants that may be explained by the more equipped nature of the academic dental facilities and continuous education available to the academic staff. This is consistent with the findings of Ramesh Nayakar et al. [16] study which also revealed that the teaching faculty showed a better understanding of digital technology compared to private practitioners. In comparison with participants taking the Great Cairo region as governorate of practice, Alexandria and Delta/Suez Canal had significantly lower knowledge scores, while regarding perception scores, only Delta/Suez Canal showed a significant difference. This may be related to the knowledge of the more prevalent digital labs and clinics distributed in Cairo than the rest of the governorates, but, the insignificance between Upper Egypt and Great Cairo is not justified. Furthermore, participants who were practicing in rural areas had significantly lower knowledge, awareness, and perception when compared with those who were practicing in urban areas. This may be explained by the finding that there is still a significant urban–rural digital gap in digital practices [25]. Additionally, this difference between rural and urban dentists may be because dentists practicing in rural areas are not interested in digital dentistry because of its biggest obstacle, which is the product’s massive price and the unaffordable cost for patients in these areas.

Hegedus, T et al. [26] conducted an online survey on participants from 20 different countries with the most considerable numbers being from Hungary, the United States, and the United Kingdom, he concluded that the majority of dental practitioners depended on digital dentistry to fabricate their prostheses, casts, surgical guides, and clear aligners. In our study when different specialties were compared with general practitioner dentists, prosthodontists showed the highest level of knowledge and awareness among the participants, which is expected as CAD-CAM technology are mostly studied in the prosthodontics curriculum, with its main application in fabricating crown, veneers, bridges, implant prosthetics, and removable dentures [4, 5]. However, the current uses of digital dentistry are not limited to the prosthodontics field as it infiltrated almost all other specialties and it’s expected for other dental specialists to be also well informed and knowledgeable about this important technology. The lowest scores were related to pedodontists and preventive dentists, respectively. This may be because the use of digital dentistry in their practices is still not as prevalent as in the rest of the specialties. However, it demonstrates a deficiency in digital dental technology education, which needs to be addressed by the incorporation of courses and workshops in pediatric postgraduate studies, as pediatric dentistry can benefit from digital technology in fabricating various types of prostheses or orthodontic appliances. These technologies can provide pediatric patients with the best dental care possible. In addition, it has the power to encourage pediatric patients and foster a cooperative, positive attitude and behavior toward the profession [27]. Contrary to the findings of the study conducted in Switzerland [28] which concluded that younger dentists were more into digitalization, as well as a survey conducted in the United States among AirForce general dentists in 2020 [29], the current study found no differences in the level of knowledge, awareness, or perception based on the participant’s age or level of experience. This may be explained by the insufficient shift in the curriculum to integrate digital dentistry courses in Egyptian dental schools.

The current study revealed that with continuous dental education, availability of equipment, and incorporation of modern digital dental education at both the undergraduate and postgraduate levels, the overall knowledge and awareness of the practicing dentist would be improved, which would positively influence the quality of care and level of satisfaction offered to the patients. This is evident by the higher levels of knowledge and awareness among prosthodontists, academic staff, and dentists practicing in the more equipped regions compared with their colleagues. Additionally, to provide dentists with the skills they need to give their patients the best care possible, it is essential to address the knowledge gaps that exist between dentists with different specializations and work environments. This can be done by creating educational programs that are tailored to the needs of the least knowledgeable groups, whether at dental faculties or government healthcare facilities.

### Strength and limitations

The current study has some limitations. It’s based on an online survey which is known for its lower response rate than paper-based surveys [30]. Although we used an anonymous questionnaire, there is a possibility of response bias because of social desirability that may lead participants to overestimate their knowledge, awareness, or perception of digital dentistry. There is also a possibility of recall bias, as participants may have difficulty accurately recalling past experiences or levels of knowledge. In addition, participants were recruited using snowball sampling methods since this study was the first to be done to assess digital dentistry KAP among Egyptian dentists, also participation may be reluctant to the participant’s interest in the topic or the time they spend on social media. On the other hand, this is the first study to investigate the dentist’s knowledge in this important field. Also, the study sample included dentists from both genders, with varying levels of experience and working in different health sectors and governorates in Egypt. This paper should be considered a reference for future paper-based studies with larger sample sizes to confirm the findings of this study. Furthermore, future studies should aim to assess the current state of digital dentistry in Egypt including its adaptation in the diagnosis and treatments among dental practitioners, and the barriers they face using this technology.

## Conclusions

The present study indicates differences in the knowledge, awareness, and perception among dental practitioners in Egypt working in different institutions. Dentists practicing in academia showed a higher level of awareness than their colleagues in other institutions. Prosthodontists showed the highest level of knowledge among the study participants. Furthermore, Participants of Great Cairo and Upper Egypt had high knowledge, awareness, and perception levels, as well as those of urban areas. About half of the dentists in Egypt had moderate knowledge and awareness levels, while most of them had a high level of perception toward practicing digital dentistry. This study demonstrates a deficit in digital dentistry education, which needs to be addressed at both undergraduate and postgraduate levels. The introduction of sufficient theoretical and practical hands-on training programs can assist dentists in gaining the skills and knowledge required for using these technologies efficiently. Therefore, dental education programs in this important field should gain more attention from policymakers by providing adequate funding and resources for implementing these programs, especially for dentists practicing in rural areas, outside the great Cairo region, and in governmental clinics. Courses and workshops will need to be organized in different governmental, and private faculties of dentistry in different governorates.

### Supplementary Information

**Additional file 1. **

## Data Availability

The datasets used and/or analyzed during the current study are available from the corresponding author upon reasonable request.
