# Supplementary material for: Knowledge, awareness, and perception of digital dentistry among Egyptian dentists: a cross-sectional study
Source: BMC Oral Health. 2023 Dec 4;23:963. doi: 10.1186/s12903-023-03698-1 (PMC10694936; doi:10.1186/s12903-023-03698-1)
Supplement: Supplementary file 1 — Additional file 1. [file 12903_2023_3698_MOESM1_ESM.docx]

**Knowledge, Awareness, and Perception of Digital Dentistry among Egyptian Dentists: A Cross-Sectional Study**

Dear Fellow Dentists,

This questionnaire aims to assess knowledge, awareness, and perception of digital dentistry.

**Kindly note that**

- Participation is voluntary, and by filling out the forum you are consenting to participate in the study.
- Any data collected will be maintained with the research team only and only results will be shared for publication purposes.
- You will not be asked to provide any personal details (Name, phone number, or Email)
- This study was approved by Pharos university research ethics committee registration no (04-2022-11-27-3-047).

1. **Demographic Section**
2. **Type of practice**

1. Private practitioner

2. Governmental sector

3. Teaching faculty

4. Postgraduate student

1. **Gender**

1. Male

2. Female

1. **Age Group**

1. 20-29

2. 30-39

3. 40-49

4. 50-59

5. 60 or above

1. **Years of clinical experience**

1. Less than 5 years

2. 5-10 years

3. More than 10 years

1. **Governorate of practice (محافظة العمل)**

Your answer

1. **Location of your practice**

1. Urban areas

2. Rural areas

1. **You are**

1. General dental practitioner

2. Prosthodontist

3. Orthodontist

4. Oral surgeon

5. Restorative

6. Pedodontist

6. Endodontist

8. Family Dentist

9. Preventive Dentist

10. Other: Specify:

1. **Knowledge Section**
2. **Which procedure in digital technology is useful in dentistry? (Please Select all that Apply)**

1. Crown and bridge fabrication

2. Implant restorations

3. Impression making

4. Maxillofacial prosthesis

5. Surgical reconstruction

6. Smile designing

7. Invisible orthodontics

8. Other:

9. I do not know

1. **Use of CAD/CAM in dentistry**

1. Intraoral scanning

2. Digital impressions

3. Shade matching

4. Computer-aided designing (by laboratory or specialist milling center)

5. Computer-aided manufacturing (by laboratory or specialist milling center)

6. I do not know

1. **Advantages of CAD/CAM**

1. Reduced number of appointments

2. Less chair side time

3. More precise as compared to conventional methods

4. Digital data flow

5. Other: Specify:

6. I do not know

1. **Advantages of CAD/CAM in the clinical scenario**

1. Eliminates the problems associated with impression making

2. Can review your preparation and modify it at the same time

3. Immediate data transfer and retrievability of scan data at any point

4. Ease in laboratory authorization and communication

5. Accurate and precise fit of the restoration/orthodontic appliances

6. Accurate and precise orthodontic tooth movement

7. I do not know

1. **Shortcomings of the use of CAD/CAM**

1. High cost

2. Lack of infrastructure

3. Lack of knowledge

4. Prefer conventional methods

5. I do not know

**C. Awareness Section**

1. **Are you aware of the CAD-CAM technology clinical applications in dentistry?**

1. YES

2. NO

1. **Are you aware of the use of digital technology in dentistry?**

1. YES

2. NO

1. **Which of the following CAD-CAM system are you aware of?** **(Please Select all that Apply)**

1. Lava™

2. Distributed Control System (DCS) Precident

3. Chairside Economical Restoration of Esthetic Ceramic (CEREC)

4. Procera

5. None

1. **Which of the following materials are used with CAD-CAM systems?** **(Please Select all that Apply)**

1. Emax

2. Zirconia

3. Metals

4. Composite

5. I do not know

**D. Perception/Practice Section**

1. **In your opinion do you think that Digital Dentistry would have a role to play in the current COVID-19 scenario?**

1. Yes

2. No

3. Not sure

1. **Did you attend any training program or workshop on CAD/CAM?**

1. Yes

2. No

1. **Do you think there is a need to increase knowledge regarding digital dentistry during undergraduate/postgraduate courses?**

1. Yes

2. No

3. Not sure

1. **Are you interested in incorporating CAD/CAM into your regular workflow?**

1. Yes

2. No

3. Not sure

1. **Would you prefer CAD/CAM over conventional methods?**

1. Yes

2. No

3. Not sure

1. **Do you think digital dentistry would affect your clinical decision-making?**

1. Yes

2. No

3. Not sure

1. **Do you think digital dentistry would have a positive impact on our profession and would be the future of dental practice?**

1. Yes

2. No

3. Not sure

**Thank you**
